# The complete chloroplast genome sequence of the *Dioscorea esculenta* (Lour.) Burkill (Dioscoreaceae)

**DOI:** 10.1080/23802359.2020.1832593

**Published:** 2020-11-20

**Authors:** Xin Chen, Lijuan Cai, Yu Zhang, Wenzheng Su, Bicong Li, Qinghong Zhou, Qianglong Zhu

**Affiliations:** a Department of Horticulture, College of Agronomy, Jiangxi Agricultural University, Nanchang, P.R. China; b Nanchang Business College, Jiangxi Agricultural University, Nanchang, P.R. China

**Keywords:** *Dioscorea esculenta* (Lour.) Burkill, chloroplast genome, lesser yam

## Abstract

*Dioscorea esculenta* (Lour.) Burkill is an essential tuber crop with pharmacological effects in the family Dioscoreaceae. The complete chloroplast genome of *D. esculenta* was determined in this study. The total genome size is 153, 437 bp in length and demonstrates a typical quadripartite structure containing a large single copy (LSC, 83,628 bp) and a small single copy (SSC, 18,893 bp), separated by a pair of inverted repeats (IRa, IRb) of 25,458 bp. The GC content of the complete chloroplast genome sequence is 37.07%. A total of 131 genes were predicted including 86 protein-coding genes, 37 tRNA genes, and eight rRNA genes. Phylogenetic tree analysis of 25 species belonging to the genus *Dioscorea* indicated that *D. esculenta* and *D. sansibarensis* were clustered into one branch.

*Dioscorea esculenta* (Lour.) Burkill (Dioscoreaceae) is an edible tuber crop and belongs to the family Dioscoreaceae, commonly known as lesser yam (Olayemi and Ajaiyeoba 2007). The tuber of *D*. *esculenta* contains rich starchprotein and soluble sugar, thus it has been as one of the staple foods in most parts of West Africa, and has been also used as an crude drug for anti-inflammatory, anti-stress, anti-spasmodic, and antifatigue for its medical elements of allantoin, saponin and dioscin (Murugan and Mohan 2012; Lee et al. 2017). *Dioscorea* is highly species-rich (approximately 600 species) and morphologically complex genus, and their phylogenetic relationship has not been uncovered better (Magwé-Tindo et al. 2019; Darkwa et al. 2020). The chloroplast genome has smaller size and low recombination, which makes it suitable for species identification and molecular phylogenetic analyses (Govindarajulu et al. 2015). Here, we analyzed the characters of the complete chloroplast genome sequence for *D. esculenta,* to confirm its phylogenetic position and evolutionary relationship between the *D. esculenta and* other species in Dioscoreaceae family.

*Dioscorea esculenta* (YAM-256) was planted in the Yulan experimental station of Jiangxi Agricultural University (28°45′27″N, 115°50′20″E), Nanchang, China. Total DNA was extracted from fresh leaves and subjected to construct a genomic library and sequenced by HiSeq X Ten (BGI, Shenzhen, China). About 3 Gb of clean sequence data with ∼5× coverage to its nuclear genome was obtained, and 0.4 Gb of clean pair-end reads with a length of 150 bp was randomly extracted using Seqtk. The lesser yam chloroplast graft genome was *de novo* assembled using SPAdes (v 3.14.1) (Bankevich et al. 2012), BlastN (v2.7.1), and Gapcloser (v1.12-r6). Specific method is as follows: Firstly, these reads were assembled with using the Plasmidspades.py in SPAdes. Secondly, Contigs representing the chloroplast genome were then retrieved, ordered and incorporated into a single draft sequence by BlastN against the chloroplast genome of *D*. *polystachya* (NC_037716.1). Thirdly, the gaps in the chloroplast single draft sequence of were closed by using GapCloser. Finally, the complete genome sequence was annotated using CPGAVA2 (Shi et al. 2019) and manually corrected by Sequin and IGV (Thorvaldsdóttir et al. 2013).

The complete chloroplast genome of *D. esculenta* (GenBank accession number: MT818507) is 153,437 bp in length with 37.07% GC contents and exhibits a typical quadripartite structure, consisting of a pair of inverted repeat regions (IRa and IRb) with same length (25,458 bp) separated by the large single copy (LSC, 83,628 bp) and small single copy (SSC, 18,893 bp) regions. A total of 131 genes were identified, including 86 protein-coding genes, eight rRNA genes, and 37 tRNA genes; six of the protein-coding genes, six of the tRNA genes, and four rRNA genes are duplicated within the IRs. Noticeably, the start code of rpl2 is by RNA editing, and one of ycf1 is a pseudogene that has only partial fragment.

To explore the phylogenetic position and evolutionary relationship of *D. esculenta*, a phylogenetic analysis was conducted between *D. esculenta* and other 24 complete chloroplast genomes of Dioscoreaceae downloaded from the organelle genome database in NCBI using Batch Entrez, and *Colocasia esculenta* chloroplast genome as the outgroup. These sequences above were aligned using MAFFT v7.407 (Nakamura et al. 2018) and the phylogenetic tree was constructed using MEGA v10.0.4 (Kumar et al. 2018) with maximum likelihood (ML) method. The result was well-resolved and revealed that *D. esculenta* was belonged to *Dioscoreaceae* and closer to *D. sansibarensis* (Figure 1), these findings further enriched the phylogenetic relationship of the family Dioscoreaceae (Cao et al. 2018; Zhao et al. 2018; Magwé-Tindo et al. 2019).

**Figure 1.**
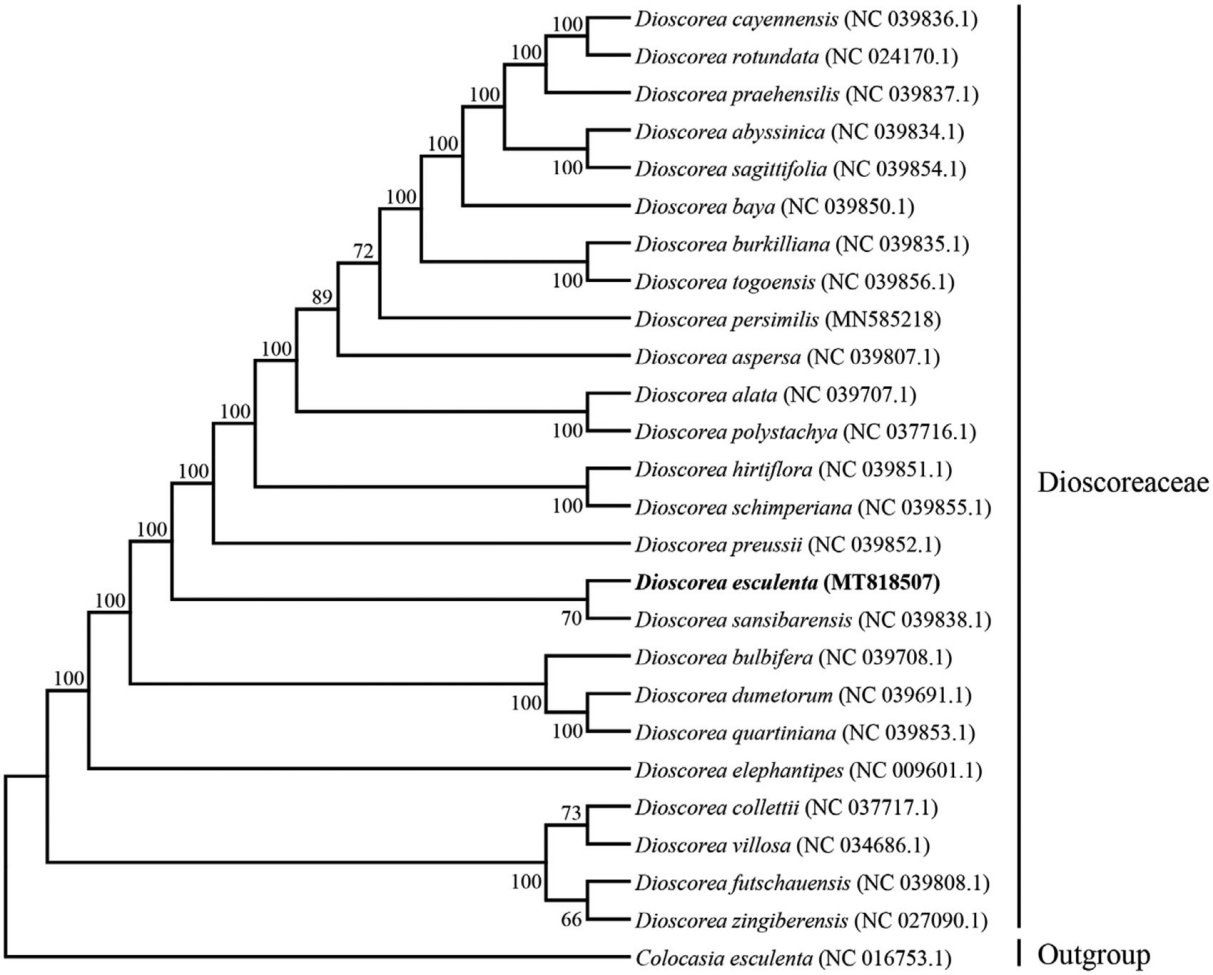
Phylogenetic tree showing the relationship between *D. esculenta* and 24 species belonging to Dioscoreaceae family, *C. esculenta* was taken as the outgroup. Phylogenetic tree was constructed based on the complete chloroplast genomes using maximum likelihood (ML) with 1000 bootstrap replicates. Numbers in each the node indicated the bootstrap support values.

## Data Availability

The data that support the findings of this study are openly available in GenBank at https://www.ncbi.nlm.nih.gov/nuccore/ MT818507.1/, reference number MT818507.
